# Peripheral inflammation levels associated with degree of advanced brain aging in schizophrenia

**DOI:** 10.3389/fpsyt.2022.966439

**Published:** 2022-08-12

**Authors:** Federica Klaus, Tanya T. Nguyen, Michael L. Thomas, Sharon C. Liou, Benchawanna Soontornniyomkij, Kyle Mitchell, Rebecca Daly, Ashley N. Sutherland, Dilip V. Jeste, Lisa T. Eyler

**Affiliations:** ^1^Department of Psychiatry, UC San Diego, La Jolla, CA, United States; ^2^VA San Diego Healthcare System, La Jolla, CA, United States; ^3^Department of Psychology, Colorado State University, Fort Collins, CO, United States; ^4^Sam and Rose Stein Institute for Research on Aging, University of California, San Diego, La Jolla, CA, United States; ^5^Department of Neurosciences, University of California, San Diego, La Jolla, CA, United States

**Keywords:** inflammation, brain, schizophrenia, aging, MRI, TNFα, negative symptoms, cognition

## Abstract

Brain structural abnormalities have been demonstrated in schizophrenia (SZ); these resemble those seen in typical aging, but are seen at younger ages. Furthermore, SZ is associated with accelerated global brain aging, as measured by brain structure-based brain predicted age difference (Brain-PAD). High heterogeneity exists in the degree of brain abnormalities in SZ, and individual differences may be related to levels of peripheral inflammation and may relate to cognitive deficits and negative symptoms. The goal of our study was to investigate the relationship between brain aging, peripheral inflammation, and symptoms of SZ. We hypothesized older brain-PAD in SZ vs. healthy comparison (HC) participants, as well as positive relationships of brain-PAD with peripheral inflammation markers and symptoms in SZ. We analyzed data from two cross-sectional studies in SZ (*n* = 26; M/F: 21/5) and HC (*n* = 28; 20/8) (22–64 years). Brain-PAD was calculated using a previously validated Gaussian process regression model applied to raw T1-weighted MRI data. Plasma levels of inflammatory biomarkers (CRP, Eotaxin, Fractalkine, IP10, IL6, IL10, ICAM1, IFNγ, MCP1, MIP1β, SAA, TNFα, VEGF, VCAM1) and cognitive and negative symptoms were assessed. We observed a higher brain-PAD in SZ vs. HC, and advanced brain age relative to chronological age was related to higher peripheral levels of TNFα in the overall group and in the SZ group; other inflammatory markers were not related to brain-PAD. Within the SZ group, we observed no association between cognitive or negative symptoms and brain-PAD. These results support our hypothesis of advanced brain aging in SZ. Furthermore, our findings on the relationship of the pro-inflammatory cytokine TNFα with higher brain-PAD of SZ are relevant to explain heterogeneity of brain ages in SZ, but we did not find strong evidence for cognitive or negative symptom relationships with brain-PAD.

## Introduction

The etiology of SZ remains unclear (1–5). Brain structural changes, such as reduced cortical thickness, have been demonstrated (6) that resemble those seen in advanced aging (7, 8). Several studies have observed physiological changes, seen throughout the body with normal aging, occurring at an earlier age in people with SZ (9–11). This observation led to the hypothesis of accelerated aging in SZ (10, 12, 13). Machine-learning techniques have been used to estimate the degree to which one's global brain age differs from one's chronological age (brain-predicted age difference or brain-PAD). Such studies have shown a brain-PAD of 3–5 years in SZ, indicating a brain with an older biological vs. chronological age (14–17). Brain-PAD has been found to increase across the disease stages of SZ, and also predicts negative and disorganized symptoms (14). Additionally, in a longitudinal study in SZ, brain-PAD increased significantly more than would be expected by chronological time during the follow-up, indicating accelerated brain aging in SZ (16). High heterogeneity exists in the degree of observed brain abnormalities (18, 19), with inflammation potentially playing an important role (20). Specifically, accelerated aging in SZ has been shown to include a chronic pro-inflammatory state of the peripheral immune system, as well as inflammation-related alterations of the brain (14–16, 21, 22).

Evidence suggests a general dysregulation in peripheral inflammation in SZ. Higher levels of peripheral pro-inflammatory mediators have been associated with altered brain morphology, negative (1), and cognitive symptoms in SZ (23–27), and also activation of central inflammatory mechanisms (28–30). Similar findings can also be observed in typical aging, which can be characterized by a chronic, low-grade inflammation (31) and behavioral symptoms that also occur at an earlier stage in SZ, such as apathy and cognitive decline in certain domains (7, 8, 32, 33). In typical aging, studies have demonstrated that structural measures of gray and white matter health in several cortical and subcortical brain areas were associated with inflammation (34–37). The frontal cortex appears particularly vulnerable to the possibly inflammation-related effects of aging (33).

In SZ, increased peripheral inflammation has been shown to be related to altered brain structure, specifically reduced cortical thickness (27, 38–40). In addition, alterations in frontal cortical thickness have been demonstrated to be related to impairment in cognition and motivation behavior, such as apathy (33, 41, 42). Notably, frontal cortical brain regions have been shown to be particularly vulnerable to inflammation (33, 41, 42). An increase of age-associated inflammatory activity in the brain in conjunction with the periphery was found in SZ (24, 43). However, there are also conflicting findings (26, 44), emphasizing the need for research into heterogeneity and subgroup identification. Additionally, the extent and nature of the inter-relationship of peripheral inflammation and brain aging and their relation to specific symptoms of SZ remains to be elucidated. Brain-PAD is a valid and reliable phenotype of aging-related changes in brain structure that, in combination with measures of peripheral biomarkers such as inflammatory mediators, has the potential to provide personalized information on brain health in mental illnesses such as SZ (45). While inflammation is intertwined with brain aging (46), the relationship between peripheral inflammation and brain-PAD in SZ has not been elucidated.

The goal of our study was to investigate the relationship between a biomarker of accelerated brain aging, peripheral inflammation, and symptoms of SZ. We focused on negative symptoms and cognitive impairments due to their clinical relevance and potential for personalized treatment approaches by identifying factors that might contribute to heterogeneity in disease presentation. We hypothesized that SZ would have an older brain-PAD than healthy comparison (HC) participants. We also expected to find positive associations between brain-PAD with peripheral inflammation markers and symptoms in SZ, such that those with older brain age compared to chronological age would show greater inflammation, poorer cognition, and worse negative symptoms.

## Methods

### Subjects

We analyzed combined data from two cross-sectional studies of patients with SZ (*n* = 26; M/F: 21/5) and HC (*n* = 28; 20/8), aged 22–64 years. People with SZ were recruited from outpatient clinics of the Veterans Affairs (VA) San Diego Healthcare System and affiliated institutions. HCs were recruited through advertisement and recruitment flyers in the community and word of mouth. No randomization occurred. Participants were screened on the phone prior to enrollment to ensure they were eligible based on the following criteria: right-handed, no history of neurological (e.g., stroke), psychiatric, or substance use disorders besides a diagnosis of SZ for the patient group, and did not have MRI (magnetic resonance imaging), contraindications (e.g., pacemaker or other implanted metallic devices, claustrophobia, or metallic dental implants). Participation involved (1) blood draw for assessment of inflammatory markers (only sub-study 1), (2) a neuropsychological and clinical assessment for the confirmation of presence (SZ) or absence (HC) of diagnoses based on the Structured Clinical Interview for the DSM-IV-TR (SCID) and Mini-International Neuropsychiatric (MINI) Interview (47, 48) and assessment of cognition and negative symptoms, and (3) a structural MRI scan. These assessments were completed on 1 day. The study was approved by the Internal Review Board at the University of California at San Diego and the UCSD Human Research Protections Program. Participant informed consent and data were acquired according to the guidelines established by the Helsinki Declaration.

### Clinical and neuropsychological assessment

Negative symptoms in SZ patients were assessed using the rater-administered Scale for Assessment of Negative Symptoms (SANS) (49) and a total score was calculated by summing the scores of the global assessment (items 8, 13, 17, 22, and 25). Positive symptoms were assessed for sample descriptive purposes using the sum of scores of global assessments of the rater-administered Scale for Assessment of Positive Symptoms (SAPS) (49) (items 7, 20, 25, and 34). These data were available from both studies. Depressive symptoms were assessed for sample descriptive purposes using the Center for Epidemiological Studies-Depression Scale (50). These data were only available from sub-study 1.

Cognition was assessed with selected subsets of the Delis-Kaplan Executive Function System (D-KEFS) (51) including Trail Making Letter-Number Sequencing, Color Word Inhibition (Switching), and Letter Fluency (FAS total) according to Palmer et al. (52). D-KEFS raw scores were converted to age-corrected t-scores to create a composite cognitive t-score. Data were available from sub-study 1.

All assessments were performed by certified raters. Due to few measurements available in the HC group (see Table 1 for details) and a research question focus on cognitive impairment and negative symptoms within the SZ group, only assessment data from participants with SZ were analyzed.

**Table 1:** Sociodemographic and clinical variables and brain age measures.

	**Healthy comparison**		**Schizophrenia**		**Test statistics**		
	***n* total sample** **(sub-study 1/2)**	**Mean (SD) or % (*n*)**	***n* total sample** **(sub-study 1/2)**	**Mean (SD) or % (*n*)**	**χ^2^/*F***	***p***	**φ/partial η^2^**
**Sociodemographic factors**							
Age (years)	28 (20/8)	42.5 (9.00)	26 (20/6)	45.1 (8.91)	*F*_(1,51)_ = 1.52	0.22	Partial η^2^*=* 0.03
Education (years)	28 (20/8)	15.2 (2.53)	26 (20/6)	11.96 (2.22)	*F*_(1,51)_ = 25.30	**<0.001**	Partial η^2^*=* 0.34
Gender (% male)	28 (20/8)	71% (20)	26 (20/6)	81% (21)	χ^2^ *=* 0.64	0.42	*φ=* 0.11
Race (% (*n*) White, African American, Asian, Asian Indian, Chinese, Filipino)	28 (20/8)	67% (19), 11% (3), 0% (0), 11% (3), 0% (0), 11% (3)	26 (20/6)	65% (17), 12% (3), 8% (2), 12% (3), 4% (1), 0% (0)	χ^2^ *=* 6.05	0.30	*φ=* 0.34
Hispanic origin	20 (20/0)	35% (7)	20 (20/0)	30% (6)	χ^2^ *=* 0.11	0.74	*φ=* 0.05
Antipsychotic medication (% Yes)	–	–	20 (20/0)	75% (15)	–	–	–
Antidepressant medication (% Yes)	–	–	20 (20/0)	85% (17)	–	–	–
Non-psychiatric medication (% Yes)	20 (20/0)	25% (5)	20 (20/0)	60 % (12)	χ^2^ *=* 5.01	**0.03**	*φ=* 0.35
**Cognitive and negative symptoms**							
CES-D total score	18 (18/0)	2.83 (2.9)	12 (12/0)	8.17 (4,2)	*F*_(1,28)_ = 17.2	**<0.001**	Partial η^2^*=* 0.38
SAPS total score^a^	14 (7/7)	0.71 (1.44)	23 (17/6)	5.22 (4.09)	*F*_(1,35)_ = 15.65	**<0.001**	Partial η^2^*=* 0.30
SANS total score^b^	14 (7/7)	0.86 (1.66)	23 (17/6)	6.61 (3.94)	*F*_(1,35)_ = 26.70	**<0.001**	Partial η^2^*=* 0.41
Cognitive composite t-score	19 (19/0)	54 (9.15)	18 (18/0)	33.4 (4.28)	*F*_(1,35)_ = 19.19	**<0.001**	Partial η^2^*=* 0.35
**Brain age measures**							
Brain age (years)^c^	26 (19/7)	37.93 (9.93)	25 (20/5)	47.1 (11.3)	*F*_(1,49)_ = 9.48	**0.003**	Partial η^2^*=* 0.20
Brain-PAD (years)^c^	26 (19/7)	−4.1 (5.6)	25 (20/5)	2.4 (7.4)	*F*_(1,48)_= 12.50	**<0.001**	Partial η^2^*=* 0.20

SANS, Scale for the Assessment of Negative Symptoms.

Sub-study included in all linear mixed models as random effect except for Cognitive composite.

a Sum of global assessments (items 7, 20, 25, and 34).

b Sum of global assessments (items 8, 13, 17, 22, and 25).

c Corrected for age.

Significant results with p-values < 0.05 are bolded.

### Peripheral inflammatory biomarkers

Findings of increased levels of peripheral inflammation in SZ vs. HC have been previously published based on a subset of the data presented here from the study that also measured inflammatory markers (53). Plasma levels of cytokine, chemokine, and vascular biomarkers (CRP, Eotaxin, Fractalkine, IP10, IL6, IL10, ICAM1, IFNγ, MCP1, MIP1β, SAA, TNFα, VEGF, VCAM1) were quantified in available samples of one sub-study (sub-study 1, *n* = 20/19 (HC/SZ)). Non-fasting blood samples were drawn by a certified phlebotomist into ethylenediaminetetraacetic acid (EDTA)-treated vacutainers. For details on analysis procedures see (54, 55), results on this dataset were published in (53). Intra-assay variability was <5% for all assays, except for IL-10 (intra-assay CV 6%). No sample showed biomarker levels below the detection limits. Results were FDR-adjusted (56) (p.adj) in all analyses including inflammatory markers.

### MR image acquisition

MRI was performed at the UCSD Keck Center for Functional MRI on a General Electric (GE) Discovery MR750 3.0 Tesla whole-body imaging system with a Nova 32-channel phased-array head coil using the Human Connectome Project Lifespan protocol sequences (57).

Anatomical scans were acquired using a T1-weighted spoiled gradient echo sequence with fast and prospective motion correction imaging options (TR = 7.4 ms; TI = 1,060 ms; TE = 2.3 ms; flip angle = 8°; FOV = 25.6 cm; matrix size = 320 × 320; slices = 204; slice thickness = 0.8 mm; slice spacing = 0 mm). Structural images were acquired in sagittal planes parallel to the intercommissural line in an interleaved manner.

### Data processing and analysis

#### Brain age calculations

Brain-predicted age was calculated using “BrainAgeR” a validated predictive modeling approach based on Gaussian process regression applied to voxel-level raw T1-weighted MRI data (based on a training set of 3377 HC aged 18–92 years from seven publicly-available datasets) (45, 58). In brief, a model that best predicted chronological age based on voxel-level brain features in the training set was applied to the current MRI data resulting in a predicted brain age for each person. Brain-PAD was then calculated as the difference between predicted brain age minus chronological age (positive values indicate an older brain age than chronological age and negative values indicate a younger brain age than chronological age).

In order to test if the previously validated model would fit our data well, we investigated the relationship between chronological age and predicted brain age and found a strong correlation between predicted brain age and chronological age in the overall group [r_49_ = 0.77, *p* < 0.001] as well as in the HC [*r*_24_ = 0.83, *p* < 0.001] and SZ [r_23_ = 0.76, *p* < 0.001] groups separately, indicating accuracy of the BrainAgeR method (Supplementary Figure S1). This is also supported by a mean absolute error (MAE) for the overall group of 5.83 years and separately for the HC group 5.65 years and for the SZ group 6.02 years, which are only slightly higher than the MAE from the original validation sample (*n* = 857), which was 3.93 years.

Furthermore, as common in studies of brain-PAD, there was a positive correlation of age with brain-PAD, therefore we used age as a covariate in all models with brain-PAD or brain age to account for age-related bias, that is, an underestimation of brain age in older individuals and vice versa (59–62). For illustrative purposes, relative brain-PAD (Residual) was calculated as the residual from the regression of Brain Age-predicted age on chronological age and this value was used in the Figures.

#### Statistical analysis

Demographic categorical variables were analyzed with χ^2^ tests and linear mixed-effects models were used to investigate group differences. Due to potential clustering of data within the two sub-studies that were combined, sub-study was included as a random intercept effect in all analyses except for those where only data from one sub-study were available (inflammatory markers and cognitive assessments).

Fixed effects were diagnostic group (in all analyses except in analyses of the SZ group only), and age (in all analyses of brain age and brain-PAD), and respective factors of interest (i.e., biomarkers, symptom measurements, and brain-PAD).

All available data was included in the analyses. Analyses of clinical symptoms were only conducted within the SZ group due to the absence of psychopathological symptoms in HC. Analysis involving several inflammatory marker analyses were adjusted for multiple testing with FDR (56) (denoted as p.adj).

Details on model parameters and estimates can be found in Supplementary Table S1.

Level of statistical significance was set at *p* < 0.05. Statistical analyses were computed with SPSS version 25 (IBM Corp., SPSS Inc., Chicago IL, USA), RStudio version 1.2.5001 (RStudio, Inc.) and GraphPad Prism software 8 (GraphPad Software Inc.). Estimates of effect sizes for chi-square tests are given as phi (φ)^2^ and for linear mixed models as partial eta squared (partial η^2^). Because of the lack of clarity in the field on how to calculate effect sizes in linear mixed models, partial η^2^ was calculated using general linear models with the same parameters as the linear mixed models and sub-study as an additional fixed effect covariate (instead of random effect). The results did not differ between the two model approaches.

## Results

### Demographics and sample characteristics, clinical symptoms, and brain age measurements

See Table 1.

### Brain-PAD is higher in SZ vs. HC indicating an older brain than expected based on the chronological age

**Figure 1:**
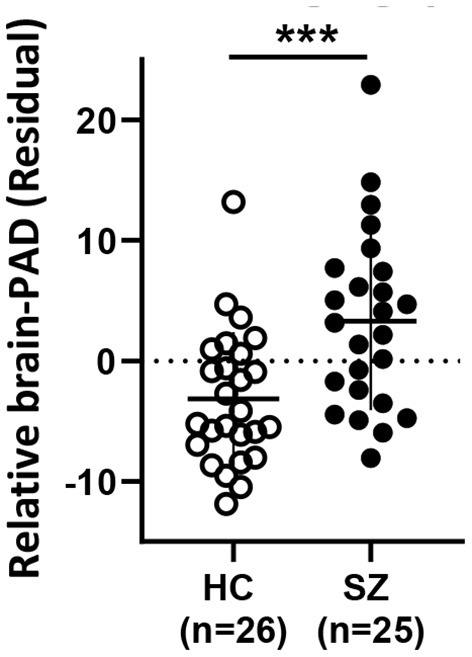
Relative Brain-PAD (residual from the regression of Brain-PAD age) differs between HC and SZ. Mean ± SD. HC: healthy controls, SZ: schizophrenia. *** *p* < 0.001.

Brain-PAD was significantly higher in SZ vs. HC, indicating a relatively younger brain than expected by chronological age in HC and an older brain than expected in SZ (see Table 1; Figure 1). Details on model parameters and estimates can be found in Supplementary Table S1.

### Higher levels of peripheral tumor necrosis factor alpha are associated with higher brain-PAD in SZ

Findings of increased levels of peripheral inflammation in SZ vs. HC have been previously published on this data set (53).

**Figure 2:**
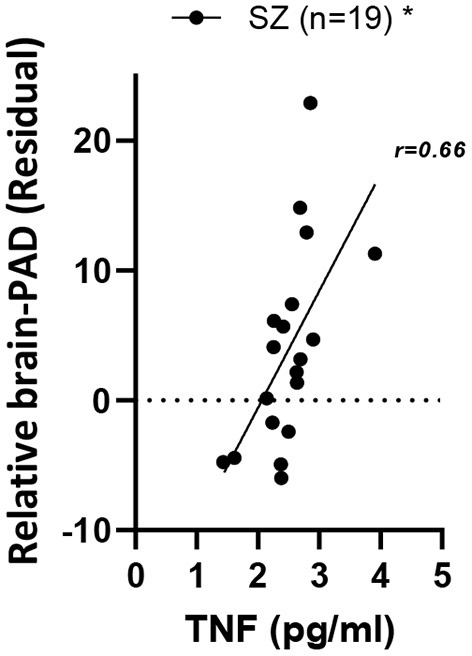
Relationship of relative brain-PAD with peripheral TNFα in SZ. SZ, schizophrenia; TNFα, Tumor necrosis factor alpha. *p < 0.05.

Regarding the relationship of brain-PAD to peripheral inflammatory markers, a significant positive relationship of brain-PAD was present only with TNFα [*F*_1,34_ = 11.7, p.adj = 0.03, η^2^ = 0.37] in the overall group. Within the SZ group, a positive relationship between TNFα and brain-PAD remained present [main effect of TNFα: *F*_1,16_ = 8.3, *p* = 0.01, η^2^ = 0.26] (Figure 2). As a follow-up, we also analyzed the HC group separately, where the same directionality of the relationship was visible, but did not reach significance [*F*_1,16_ = 2.36, *p* = 0.14, η^2^ = 0.13]. The relationship was not significantly stronger in the SZ vs. HC group; in the overall group, the diagnostic group-by-TNFα interaction was not significant [*F*_1,33_=0.55, *p* = 0.46, η^2^ = 0.02].

Details on model parameters and estimates can be found in Supplementary Table S1.

### Negative or cognitive symptoms are not associated with brain-PAD in SZ

Within the subset of the SZ group that had clinical variables available, no significant main effects of either negative symptoms [*F*_1,19_ = 1.53, *p* = 0.23, η^2^ = 0.08] or cognitive symptoms [*F*_1,15_ = 0.81, *p* = 0.38, η^2^ = 0.05] with brain-PAD were present.

Details on model parameters and estimates can be found in Supplementary Table S1.

## Discussion

This study examined the relationship between brain age based on brain structure, peripheral inflammation, and clinical symptoms in SZ. Overall, several significant results emerged; specifically, higher brain-PAD in patients with SZ compared to HC was in line with our hypothesis that participants with SZ have an older brain than expected based on the chronological age. These findings are in line with the recent literature indicating an accelerated brain aging in SZ that is reflected in structural brain changes (7, 14–17).

In line with our hypothesis of an involvement of inflammatory processes in advanced brain aging in SZ (20), we found that patients with higher peripheral levels of TNFα had more advanced brain age. We observed this relationship in the overall group and in the SZ group separately. The relationship was not significant in the HC group only, albeit in the same direction, and there was no significant group-by-TNFα interaction. While these findings might be due to the small sample size, they might also indicate that the effect is not unique to the SZ group, but also applies to HC, indicating that inflammation is a general risk factor for advanced brain aging (63). Consistent with these findings, a study of brain age in older adults with and without diabetes found in all participants an association of higher brain-PAD with higher TNFα levels, lower verbal fluency and more depressive symptoms (64), and a study in healthy adult volunteers found that the administration of an anti-inflammatory drug resulted in a temporary reduction of brain-PAD (65), supporting the role of peripheral TNFα in aging-related pro-inflammatory alterations of brain structure and processes. Since effect sizes in the SZ group were larger compared to the HC group it suggests that inflammation might play a somewhat larger role in brain aging in the context of serious mental illness. Regarding the other inflammatory biomarkers that we investigated, we found no associations with brain-PAD, which suggests that future studies on the relationship of inflammatory biomarkers with brain-PAD might want to focus on a wider array of pro-and anti-inflammatory markers with appropriate sample size and potentially also investigate their inter-relationship and sub-groups, to identify patterns that are missed with single-marker analyses.

Within the SZ group, our hypothesis of a significant positive association between clinical symptoms and brain-PAD was not supported. Previous studies have found brain-PAD to be predictive of belonging to the SZ vs. HC group, of reduced global functioning and of the presence of disorganized thinking and negative symptoms in SZ (14, 16). Furthermore, a weak, positive effect for negative symptom severity on brain-PAD was described in a large multi-cohort study (44). A potential explanation for our unexpected negative result besides sample size is the high heterogeneity of brain abnormality and clinical symptom presentation in SZ (18, 19).

There are several limitations to our study. One limitation is the small sample size, which was further reduced due to availability of certain measurements only in one of the two included studies. Inclusion of study as random effect due to possible study differences further reduced power and the small sample size might also be responsible for the absence of significant effects that otherwise could have been observed. While the BrainAgeR algorithm performed well for the age range of the participants of this study, caution has to be exerted when generalizing this approach to older people. The age-dependence of brain age and a potential domination of comparisons with outcome measures by true age rather than the brain aging process were accounted for in this study by including age as covariate in all analyses of brain age; however, more sophisticated mathematical approaches have also been proposed (59–62). Another limitation of a brain age-based approach is that it is a global summary measure that does not give information on the particular contribution of individual brain regions. Techniques to explore the driving features of the brain age output and to reduce the age bias are currently being developed (66). Another limitation is that no stratification approach was used to match sex of participants during recruitment, resulting in more male than female participants, which reduces generalizability to female patients with SZ. We also had no information on treatment onset, disease duration or duration of untreated illness and only limited data on medication use and dose and our results might not generalize to a population which does not take medication. Previous findings on brain age based on brain structural features have however shown that a higher brain-PAD in SZ was not driven by clinical characteristics such as illness duration, symptom severity or antipsychotic use and dose (6, 44, 67). The higher use of non-psychiatric medication in SZ vs. HC participants observed in our study might be an inherent feature of SZ with underlying biological mechanisms beyond environmental and lifestyle factors and impact on the brain (68). Future studies on the effect of psychiatric and non-psychiatric medication on brain age are warranted, particularly including patients with chronic SZ who are not receiving antipsychotic treatment, in order to disentangle the possible contribution of acute vs. long-term medication treatment to altered brain age in SZ.

In summary, our results support our hypothesis of advanced brain aging in SZ vs. HC as reflected in a larger, positive brain-PAD in SZ. Furthermore, our finding on the relationship of levels of the cytokine TNFα and brain-PAD in the SZ group support the hypothesis of an association between higher peripheral inflammation and older-than-chronological brain features, which is relevant to understand and explain heterogeneity of brain ages and clinical correlates in SZ. Our findings highlight the need to further address within-disorder heterogeneity potentially defined by subgroups based on brain structural and functional alterations and to characterize immune-related brain alterations and effects on brain age. Future research focusing on longitudinal data and the inclusion of multimodal imaging modalities will allow one to distinguish cohort effects from developmental changes, to account better for heterogeneity within a disease such as SZ, and potentially also be more sensitivity to interventions that modify disease-related aging processes with potential for clinical use (69).

## Data availability statement

The datasets presented in this article are not readily available, but the data that support the findings of this study are available upon reasonable request from the corresponding author and after filing an institutional data sharing agreement. The data are not publicly available to protect the privacy of research participants. Requests to access the datasets should be directed to FK, fklaus@health.ucsd.edu.

## Author contributions

FK, LE, TN, and MT: conception and design of study. TN, MT, SL, BS, AS, and LE: acquisition of data. FK, SL, KM, BS, and LE: analysis of data. FK and LE: drafting the manuscript. FK, LE, TN, MT, KM, AS, and DJ: revising the manuscript. All authors contributed to the article and approved the submitted version.

## Funding

This study was supported by Early Postdoc Mobility Fellowship of the Swiss National Science Foundation (SNSF) (Grant Number: P2ZHP3_181506) (FK), Novartis Foundation for Medical-Biological Research Fellowship (FK), NIMH 2 R01 MH094151-08 (DJ) (PI), the Sam and Rose Stein Institute for Research on Aging (DJ), and Desert-Pacific Mental Illness Research, Education, and Clinical Center Pala Pilot grant (TN).

## Conflict of interest

This study received funding from the Novartis Foundation for Medical-Biological Research. The funder was not involved in the study design, collection, analysis, interpretation of data, the writing of this article or the decision to submit it for publication.

## Publisher's note

All claims expressed in this article are solely those of the authors and do not necessarily represent those of their affiliated organizations, or those of the publisher, the editors and the reviewers. Any product that may be evaluated in this article, or claim that may be made by its manufacturer, is not guaranteed or endorsed by the publisher.

## Abbreviations

Brain-PAD: Brain predicted age difference
CES-D: Center for Epidemiological Studies-Depression Scale
CRP: C-reactie Protein
DKEFS: Delis-Kaplan Executive Function System
EDTA: Ethylenediaminetetraacetic acid
F: Female
FDR: False discovery rate
FOV: Field of view
HC: Healthy comparison
ICAM1: Intercellular adhesion molecule 1
IFNγ: Interferon gamma
IL: Interleukin
IP-10: Interferon-γ-inducible protein-10
M: Male
MAE: Mean absolute error
MCP1: Monocyte chemoattractant protein-1
MIP1β: Macrophage inflammatory protein 1 beta
MRI: Magnetic resonance imaging
SAA: Serum amyloid A
SANS: Scale for Assessment of Negative Symptoms
SAPS: Scale for Assessment of Positive Symptoms
SZ: Schizophrenia
TE: Echo time
TI: Inversion time
TNFα: Tumor Necrosis Factor alpha
TR: Repetition time
VCAM1: Vascular cell adhesion molecule 1
VCAM1: Vascular cell adhesion molecule 1
VEGF: Vascular endothelial growth factor.
