# Supplementary material for: Peripheral inflammation levels associated with degree of advanced brain aging in schizophrenia
Source: Front Psychiatry. 2022 Aug 12;13:966439. doi: 10.3389/fpsyt.2022.966439 (PMC9412908; doi:10.3389/fpsyt.2022.966439)
Supplement: Supplementary file 1 [file Table_1.DOCX]

Supplementary Material

## Supplementary Data

**Supplementary Figure S1)** Brainage correlates with age in HC and SZ group. Mean +/- SD. *HC: healthy controls, SZ: schizophrenia. * p < 0.05, ** p < 0.01, *** p < 0.001*

**Supplementary Table S1: Linear Mixed Models Parameters and Statistics**

*SANS: Scale for the Assessment of Negative Symptoms.*

*Study included in all linear mixed models on the overall group as random effect except for analysis of TNFα and cognitive composite (only data from one study).*

*Health control (HC) group was used as reference group.*

| **Linear Mixed Models Testing Diagnostic Group Effect with Covariates** | | | | | | | | | | |
| --- | --- | --- | --- | --- | --- | --- | --- | --- | --- | --- |
|  |  |  |  |  |  |  |  |  |  |  |
|  |  |  |  |  |  |  |  |  |  |  |
| **Brain-PAD** |  |  |  |  |  |  |  |  |  |  |
|  |  | **Group** | | | | | | | | |
|  |  | Numerator df | Denominator df | F | Sig. | Estimate | Std. Error | t | 95% Confidence Interval | |
|  |  |  |  |  |  |  |  |  | Lower Bound | Upper Bound |
| **Covariate: Age** |  |  |  |  |  |  |  |  |  |  |
|  |  | 1 | 47.89 | 12.46 | 0.00 | -0.05 | 0.11 | -0.44 | -0.26 | 0.17 |
|  |  |  |  |  |  |  |  |  |  |  |
|  |  |  |  |  |  |  |  |  |  |  |
| **Linear Mixed Models Testing TNFα Effect with Covariates** | | | | | | | | | | |
|  |  |  |  |  |  |  |  |  |  |  |
| **Brain-PAD** |  |  |  |  |  |  |  |  |  |  |
|  |  | **TNFα** | | | | | | | | |
|  |  | Numerator df | Denominator df | F | Sig. | Estimate | Std. Error | t | 95% Confidence Interval | |
|  |  |  |  |  |  |  |  |  | Lower Bound | Upper Bound |
| **Covariate: Age** |  |  |  |  |  |  |  |  |  |  |
|  |  | 1 | 34 | 11.70 | 0.00 | 7.63 | 2.23 | 3.42 | 3.09 | 12.16 |
|  |  |  |  |  |  |  |  |  |  |  |
|  |  |  | | | | | | | | |
| **Covariates: Age, Group , Group*TNFα** | |  |  |  |  |  |  |  |  | |
|  |  | 1 | 33 | 9.74 | 0.00 | 8.93 | 2.85 | 3.13 | 3.12 | 14.73 |
|  | Group * TNFα | 1 | 33 | 0.55 | 0.46 | -3.42 | 4.63 | -0.74 | -12.83 | 5.99 |
|  |  |  | | | | | | | | |
|  |  |  |  |  |  |  |  |  |  | |
| **Brain-PAD – HC only** |  |  |  |  |  |  |  |  |  |  |
|  |  | **TNFα** | | | | | | | | |
|  |  | Numerator df | Denominator df | F | Sig. | Estimate | Std. Error | t | 95% Confidence Interval | |
|  |  |  |  |  |  |  |  |  | Lower Bound | Upper Bound |
| **Covariate: Age** |  |  |  |  |  |  |  |  |  |  |
|  |  | 1 | 16 | 2.36 | 0.14 | 5.50 | 3.58 | 1.54 | -2.09 | 13.08 |
|  |  |  |  |  |  |  |  |  |  |  |
|  |  |  |  |  |  |  |  |  |  |  |
| **Brain-PAD – SZ only** |  |  |  |  |  |  |  |  |  |  |
|  |  | **TNFα** | | | | | | | | |
|  |  | Numerator df | Denominator df | F | Sig. | Estimate | Std. Error | t | 95% Confidence Interval | |
|  |  |  |  |  |  |  |  |  | Lower Bound | Upper Bound |
| **Covariate: Age** |  |  |  |  |  |  |  |  |  |  |
|  |  | 1 | 16 | 8.28 | 0.01 | 8.62 | 3.00 | 2.88 | 2.27 | 14.98 |
|  |  |  |  |  |  |  |  |  |  |  |
|  |  |  |  |  |  |  |  |  |  |  |
|  |  |  |  |  |  |  |  |  |  |  |
|  |  |  |  |  |  |  |  |  |  |  |
| **Linear Mixed Models Testing Cognitive Symptom Effect with Covariates - SZ only** | | | | | | | | | | |
|  |  |  |  |  |  |  |  |  |  |  |
| **Brain-PAD** |  |  |  |  |  |  |  |  |  |  |
|  |  | **Cognitive Composite** | | | | | | | | |
|  |  | Numerator df | Denominator df | F | Sig. | Estimate | Std. Error | t | 95% Confidence Interval | |
|  |  |  |  |  |  |  |  |  | Lower Bound | Upper Bound |
| **Covariate: Age** |  |  |  |  |  |  |  |  |  |  |
|  |  | 1 | 15 | 0.81 | 0.38 | -0.10 | 0.11 | -0.90 | -0.32 | 0.13 |
|  |  |  |  |  |  |  |  |  |  |  |
|  |  |  |  |  |  |  |  |  |  |  |
|  |  |  |  |  |  |  |  |  |  |  |
| **Linear Mixed Models Testing Negative Symptom Effect with Covariates - SZ only** | | | | | | | | | | |
|  |  |  |  |  |  |  |  |  |  |  |
| **Brain-PAD** |  |  |  |  |  |  |  |  |  |  |
|  |  | **Negative Symptoms (SANS)** | | | | | | | | |
|  |  | Numerator df | Denominator df | F | Sig. | Estimate | Std. Error | t | 95% Confidence Interval | |
|  |  |  |  |  |  |  |  |  | Lower Bound | Upper Bound |
| **Covariate: Age** |  |  |  |  |  |  |  |  |  |  |
|  |  |  |  |  |  |  |  |  |  |  |
|  |  | 1 | 19 | 1.53 | 0.23 | 0.58 | 0.47 | 1.24 | -0.40 | 1.56 |
|  |  |  |  |  |  |  |  |  |  |  |
|  |  |  |  |  |  |  |  |  |  |  |
|  |  |  |  |  |  |  |  |  |  |  |
